# Integrative Analyses of Transcriptome Sequencing Identify Functional miRNAs in the Chicken Embryo Fibroblasts Cells Infected With Reticuloendotheliosis Virus

**DOI:** 10.3389/fgene.2018.00340

**Published:** 2018-08-29

**Authors:** Jie Zhai, Chang Gao, Lisheng Fu, Long Jing, Shengyuan Dang, Shimin Zheng

**Affiliations:** Department of Pathophysiology, College of Veterinary Medicine, Northeast Agricultural University, Harbin, China

**Keywords:** REV, CEF, high-throughput sequencing, metabolism, cell cycle, apoptosis, mTOR signaling pathway

## Abstract

In this study, we found a much higher proportion of reticuloendotheliosis virus (REV) infected chicken embryo fibroblasts (CEF) were in active cell division phase than that of control cells which indicated that REV can affect the fate of CEF. So, we performed high-throughput sequencing and transcriptomic analysis to identify functional miRNAs, in order to figure out the possible mechanism in the interaction of REV with CEF. In total, 50 differentially expressed miRNAs (DEmiRNAs) were identified. Then target genes of DEmiRNAs were predicted and identified by transcriptome profile results. Kyoto Encyclopedia of Genes and Genomes (KEGG) enrichment were conducted to analyze the identified target genes of miRNAs which showed that metabolism, cell cycle, and apoptosis were the most related pathways involved in infection of REV. We analyzed the genes related to cell cycle which indicated that CyclinD1-CDK6 complex played an important role in regulating the transition of the cell cycle from G1 phase to S phase during REV infection. Fluorescence microscope identification showed that REV inhibited the apoptosis of CEF which was in accordance with transcriptome results. A novel miRNA, named novel-72 was found, KEGG analysis was conducted to predict the biological function of its target genes which showed that those target genes were significantly enriched in mTOR signaling pathway and functioned to promote cell cycle and cell growth during the REV infection. In conclusion, REV could induce the up-regulation of cell metabolism, cell cycle and mTOR signaling pathway while inhibit apoptosis of the cell.

## Introduction

Reticuloendotheliosis virus (REVs), as a member of the genus *Gammaretrovirus* in the family *Retroviridae* (Coffin, 1996), is a C-type avian retrovirus which can cause immunosuppression, runting disease, and lymphoma in a variety of avian hosts of chickens, turkeys, ducks, geese (Lin et al., 2009), peafowl, mallard (Jiang et al., 2014), and some other bird species (Barbacid et al., 1979; Bohls et al., 2006; Wang et al., 2012). Immunosuppression might lead to secondary infections which may aggravate the severity of the disease and co-infection of REVs with other bird pathogens (Bao et al., 2015), an especially immunosuppressive virus such as Marek's Disease Virus (MDV) (Dong et al., 2015) which cause dramatic damages to the poultry industry. REVs are worldwide distributed, and several new species were identified recently (Zhai et al., 2016), which may cause significant damage to the avian industry and even present threats to human health.

REVs are comprised of defective REV-T (Hoelzer et al., 1979, 1980) and non-defective REV (Chen et al., 1987). The genome of non-defective REV is about 9.0 kb in length consisting of a gag group-specific antigen (gag), polymerase (pol), envelope genes (env), and long terminal repeats (LTRs) (Hu et al., 1981; Niewiadomska and Gifford, 2013). The genome of REV-T was confirmed to be derived from non-defective REV (Rice et al., 1982; Wilhelmsen et al., 1984), which includes LTRs, parts of the gag, pol, env genes, and an additional segment about 1.5 kb, termed v-rel (Chen and Temin, 1982; Stephens et al., 1983). V-rel was proposed to be the oncogene of REV, which may cause cancer to its hosts with the assistant of other defective genes (Wilhelmsen et al., 1984). Non-defective REV could also induce lymphoma in its hosts. It has been reported that the acute death of pigeons with a histopathological test of tumor-like lesions in multiple organs which finally identified to be non-defective REV infection (Zhai et al., 2016). The tumorigenesis induced by REV reveals to be a complex mechanism. Lymphomas not only may cause death of its host but also induce immunosuppression which increases its host susceptibility to concurrent or secondary bacterial or viral infections (Jiang et al., 2014). Unveiling the interaction mechanism of REV with its host may give us a different perspective of tumorigenesis and may also help to develop novel therapies against REV infections. However, to date, the mechanisms of oncogenesis induced by REV remained to be elucidated.

Recently, studies have shown that miRNAs play as key mediators in a number of biological processes inducing oncogenesis (Akcakaya et al., 2011; Musilova and Mraz, 2015; Xiong et al., 2016; Yao et al., 2017). Yao et al. have reported that v-rel induced the overexpression of miR-155 by direct binding to NF-κB binding sites, indicating that REV-T induced transformation is mediated by the activation of NF-κB targets (Yao et al., 2017). Using high-throughput sequencing, Yu et al. (2017) have identified miRNAs that are responsible for the upregulation of proto-oncogene, and carcinogenic cytokines in chickens upon REV infections. In this study, we identified that REV can promote the progression of the cell cycle of CEF which indicated that REV can obviously affect the fate of CEF cell. Then, in order to identify the potential functions of miRNAs in CEF cells with the infection of REVs, CEF cells were infected with a tenth generation of REVs and were applied to profile both miRNA and mRNAs using high-throughput sequencing. Additionally apoptosis of CEF cells were processed to assist unveiling the functions of miRNAs and genes that were important in the interaction of REV with CEF. Our experiments paved the way to understand the pathogenesis of REV and the mechanisms of virus-host interactions.

## Materials and methods

### Ethics statement

All animal experimental procedures were approved [SYXK (Hei) 2011022] by the Animal Ethics Committee of Harbin Veterinary Research Institute of the Chinese Academy of Agricultural Sciences according to “the Australian National Health and Medical Research Council's Australian Code of Practice for the Care and Use of Animals for Scientific Purposes” and were performed according to institutional guidelines.

### Primary chicken embryo fibroblasts (CEFs) preparation

CEFs were derived from 10-day-old SPF chicken embryos which were obtained from the Experimental Animal Center of Harbin Veterinary Research Institute (HVRI), Chinese Academy of Agricultural Sciences (CAAS), China. CEFs were diluted to 1 × 10^6^ cells/ml and were cultured in Dulbecco's modified Eagle Medium (Thermo Fisher Scientific, 12100-038) containing 10% fetal bovine serum (Thermo Fisher Scientific, 12483-012) with 100 μg/ml penicillin and streptomycin for 20 cell culture bottles (25 cm^2^) at 37°C, 5% CO_2_. When cells grow to 80% confluency, 10 (one for microRNA detection, three for transcriptome analysis, three for ultrastructure identification, and the other three for cell cycle and apoptosis analysis) bottles of cells were incubated either with DMEM as controls or with 10^−5.62^ TCID_50_ of the tenth generation REV (VB). Then CEFs were cultured at 37°C, 5% CO_2_ for 3 days and were collected for testing.

### Cell cycle analysis

Flow Cytometry was applied to analyze the cell cycle. Cells were collected by the pancreatic enzyme and washed by PBS (pH = 7.2) for three times, then the samples were prepared by using Cell Cycle Kit (Beyotime Biotechnology, C1052) and analyzed by Accuri C6 (BD Biosciences, New York, America). The analyzed documents were processed by ModFit (Verity Software House, GA, America) to identify the cell cycle dynamic.

### RNA extraction and PCR amplification

Total RNA was extracted using TRIzol reagent (Invitrogen, 15596-018) according to the manufacturer's instructions. In addition, the RNase-free DNase I (Takara, 2270A) was used for removing genomic DNA contaminations from the extracted RNAs. RNA integrity was evaluated using 1% agarose gel stained with ethidium bromide (EB). Thereafter, the quality and quantity of RNA were assessed using NanoPhotometer® spectrophotometer (IMPLEN, CA, USA) and Agilent 2100 Bioanalyzer (Agilent Technologies, CA, USA). The RNA was used only when the RNA integrity number (RIN) is >8.0.

The REV RNA of the nine samples was detected by reverse transcription (RT)-polymerase chain reaction (PCR) with amplification of the viral long terminal repeat (LTR) region. Primers were designed using Primer 5.0 software (PREMIER Biosoft, CA, USA) and were listed below:

F: 5′-ATCCAATCACGAGCAAACACG-3′,

R: 5′-GCCAGCCTACACCACGAACAAAAT-3′.

The PCR products were sequenced by Sangon Biotech (Shanghai, China).

### Library construction and sequencing

A total of 3 μg RNAs per sample were used as input material for the small RNA libraries. Sequencing libraries were generated using NEBNext® Multiplex Small RNA Library Prep Set for Illumina® (NEB, USA.) following manufacturer's instructions, and index codes were added to attribute sequences to each sample. Briefly, NEB 3′ SR Adaptor was directly ligated to 3′ end of miRNA, siRNA, and piRNA. After the 3′ ligation reaction, the SR RT Primers were hybridized to the excess of 3′ SR Adaptor (that remained free after the 3′ ligation reaction) and transformed the single-stranded DNA adaptor into a double-stranded DNA (dsDNA). This step is important to prevent the adaptor-dimer formation. In addition, dsDNAs are not substrates for ligation mediated by T4 RNA Ligase 1 and therefore do not ligate to the 5′ SR Adaptor in the subsequent ligation step. The 5′ ends adapter was ligated to 5′ ends of miRNAs, siRNA, and piRNA. The first strand cDNA was synthesized using M-MuLV Reverse Transcriptase (RNase H) (NEB, M0253S). PCR amplification was performed using LongAmp Taq 2 × Master Mix (NEB, M0287S), SR Primer for Illumina and index (X) primer. PCR products were purified on an 8% agarose gel (100 V, 80 min). DNA fragments corresponding to 140–160 bp (the length of small noncoding RNA plus the 3′ and 5′ adaptors) were recovered and dissolved in 8 μl elution buffer. At last, library quality was assessed on the Agilent Bioanalyzer 2100 system using DNA High Sensitivity Chips. Then the clustering of the index-coded samples was performed on a cBot Cluster Generation System using TruSeq SR Cluster Kit v3-cBot-HS (Illumina, China) according to the manufacturer's instructions. After cluster generation, the library preparations were sequenced on an Illumina Hiseq 2500/2000 platform (Illumina) and 50 bp single-end reads were generated. The raw data have been submitted to Sequence Read Archive (https://www.ncbi.nlm.nih.gov/sra/), and the SRA accession is SRP133026.

While a total amount of 3 μg RNA per sample was used as input material for the RNA sample preparations. Then, mRNAs were purified from total RNA using poly-T oligo-attached magnetic beads. Fragmentations were carried out using divalent cations under elevated temperature in NEBNext First-Strand Synthesis Reaction Buffer (5×). The first strand cDNA was synthesized using random hexamer primer and M-MuLV Reverse Transcriptase (RNase H). The second strand cDNA synthesis was subsequently performed using DNA Polymerase I and RNase H. Remaining overhangs were converted into blunt ends via exonuclease/polymerase activities. After the adenylation of 3′ ends of DNA fragments, NEBNext Adaptor with hairpin loop structure was ligated for hybridization. In order to select cDNA fragments of preferentially 150–200 bp in length, the library fragments were purified with AMPure XP system (Beckman Coulter, Beverly, USA). Then 3 μl USER Enzyme (NEB, M5505S) was used for size-selected and adaptor-ligated cDNA at 37°C for 15 min followed by 5 min at 95°C. Then PCR was performed with Phusion High-Fidelity DNA polymerase, Universal PCR primers, and Index (X) Primer. Lastly, PCR products were purified (AMPure XP system), and library quality was assessed on the Agilent Bioanalyzer 2100 system. The clustering of the index-coded samples was performed on a cBot Cluster Generation System using TruSeq PE Cluster Kit v3-cBot-HS (Illumina) according to the manufacturer's instructions. After cluster generation, the library preparations were sequenced on an Illumina Hiseq platform and 150 bp paired-end reads were generated. Transcriptome sequencing data are available publicly at the Sequence Read Archive (https://www.ncbi.nlm.nih.gov/sra/) under accession number SRP132647.

### Analysis of sequencing results: mapping and differential expression

Clean reads of miRNA were obtained by removing reads containing ploy-N, with 5′ adapter contaminants, without 3′ adapter or the insert tag, containing ploy A or T or G or C, and low-quality reads (Q < 20) from raw data. The small RNA tags were mapped to the gallus reference genome (ftp://ftp.ensembl.org/pub/release-81/fasta/gallus_gallus/dna/) by Bowtie (Langmead et al., 2009). MiRBase20.0 was used as a reference, modified software mirdeep2 (Friedländer et al., 2011), and srna-tools-cli were used to obtain the potential miRNA and draw the secondary structures. Then the software miREvo (Wen et al., 2012) and mirdeep2 were integrated to predict novel miRNA through exploring the secondary structure, the Dicer cleavage site, and the minimum free energy of the small RNA tags unannotated in the former steps. Custom scripts were used to obtain the miRNA counts as well as base bias on the first position of identified miRNA with a certain length and on each position of all identified miRNA respectively. MiRNA expression levels were estimated by TPM (transcript per million) through the following criteria (Zhou et al., 2010):

Normalization formula: Normalized expression = mapped read count/Total reads × 10^6^. Differential expression analysis of two samples was performed using the DEGseq (2010) R package. *P*-value was adjusted using q value (Storey, 2003). *Q* < 0.05 and fold change ≥1.5 was set as the threshold for significant differential expression by default.

Clean reads of mRNA were obtained by removing adapter sequences, reads containing ploy-N, and low-quality sequences (*Q* < 20). Reference genome and gene model annotation files were downloaded from genome website directly. The index of the reference genome was built using Bowtie v2.2.3 and paired-end clean reads were aligned to the reference genome using TopHat v2.0.12. We selected TopHat as the mapping tool for that TopHat which can generate a database of splice junctions based on the gene model annotation file. HTSeq v0.6.1 was used to count the reads numbers mapped to each gene. And then the FPKM of each gene was calculated based on the length of the gene and reads count mapped to this gene. Differential expression analysis of two groups was performed using the DESeq R package (1.18.0). DESeq provides statistical routines for determining differential expression in digital gene expression data using a model based on the negative binomial distribution. *P*-values were adjusted using the Benjamini and Hochberg's approach for controlling the false discovery rate. Genes with an adjusted *P* < 0.05 by DESeq were assigned as differentially expressed.

### Integrative analysis of differentially expressed miRNAs (DEmiRNA) and DEmRNA

The prediction of target genes of DEmiRNAs was performed by using miRanda (Enright et al., 2003). Then predicted target genes of miRNAs were identified by DEmRNA. If predicted target genes showed a significantly different mRNA expression, those genes were identified to be the target genes of DEmiRNAs. Gene Ontology (GO) enrichment analysis of the identified target genes was implemented by the GOseq R package, in which gene length bias was corrected. GO terms with corrected *P* < 0.05 were considered significantly enriched by differential expressed genes. The GO annotations were functionally classified by WEGO software (http://wego.genomics.org.cn/) for gene function distributions. KOBAS software (https://login.kobas.co.uk/) was used to test the statistical enrichment of the identified target genes in KEGG pathways. The pathways with an FDR value ≤ 0.05 were defined as those with genes that display significant levels of differential expression.

### Cell apoptosis test

Two group of cells were prepared and washed by PBS (pH = 7.2) for three times, then cells were stained by JC-1 for 20 min, at 37°C according to the specification of Mitochondrial membrane potential assay kit (Beyotime Biotechnology, C2006). The red signal was tested by fluorescence microscope (NIKON CORPORATION, Tokyo Met., Japan) to analyze the early phase of cell apoptosis.

### Quantitative real-time PCR (RT-qPCR) validation of RNA-seq data

Twenty-two miRNAs including 15 targeted mRNAs and four genes related to mTOR signaling pathway were identified for validation using quantitative RT-qPCR. Primers were designed with Oligo 6.24 software (Molecular Biology Insights, Inc.) and were listed in Table S1. RT-qPCR reaction was performed in LightCycler® 2.0 system with the SYBR green PRC Master Mix (Takara, RR820A), and amplified in a final volume of 20 μl including 2 μl of cDNA template, 10 μl of 2 × SYBR Green Master Mix, and 1 μl of each primer (10 μmol/μl). The amplification program consisted of one cycle at 95°C for 10 s, followed by 50 cycles at 60°C for the 20 s, and 72°C for 20 s. Fluorescent signals were detected in the last step of each cycle. Melting curve analysis was performed at the end of 50 cycles to ensure proper amplification of target fragments. All RT-qPCR for each gene was performed in three biological replications, with the expression of *Gallus gallus* β-actin. The data was analyzed using the 2^−ΔΔ*Ct*^ method and were indicated as means ± SD (*n* = 9).

### Statistical analysis

All statistical analyses were conducted using IBM SPSS Statistics 20.0 (International Business Machines Corp., Armonk, New York, USA). *P* < 0.05 was considered statistically significant.

## Results

### Confirmation of REV infection model

**Figure 1:**
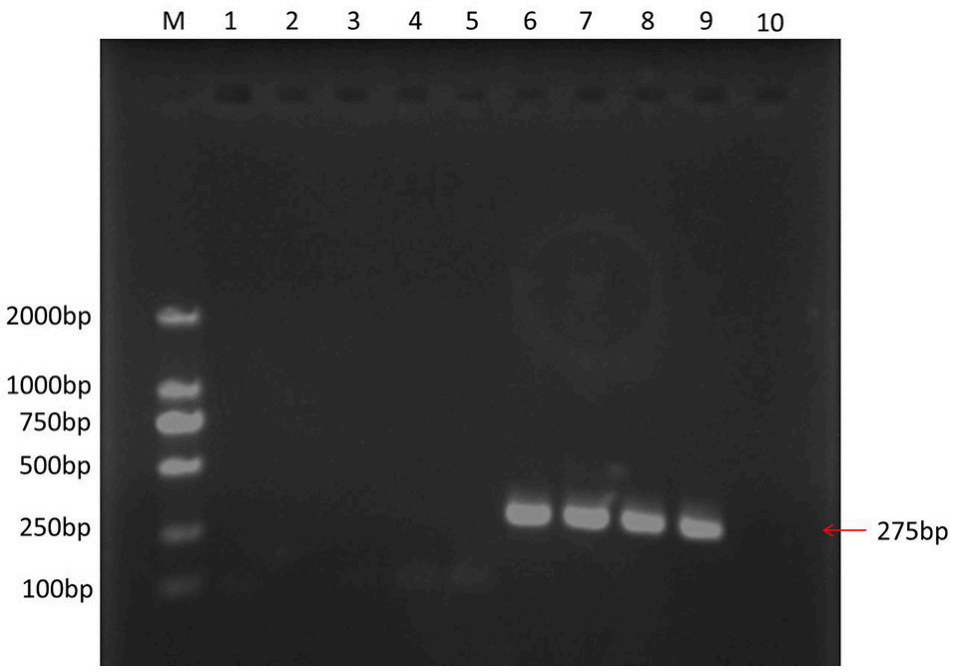
PCR result of different samples. M, marker 2000; 1, water was used as PCR temple; 2, 3, 4, and 5: control CEF cells; 6, 7, 8, and 9, VB cells.

REV infection was confirmed by RT-PCR. A 275-bp target PCR product was detected in VB samples, while none was detected in control samples (Figure 1). Upon sequencing, the PCR products showed identical to the LTR region of REV (GenBank: AY842951.1), which indicate a successful establishment of REV infection model in VB cells.

### Regulation of cell cycle

**Figure 2:**
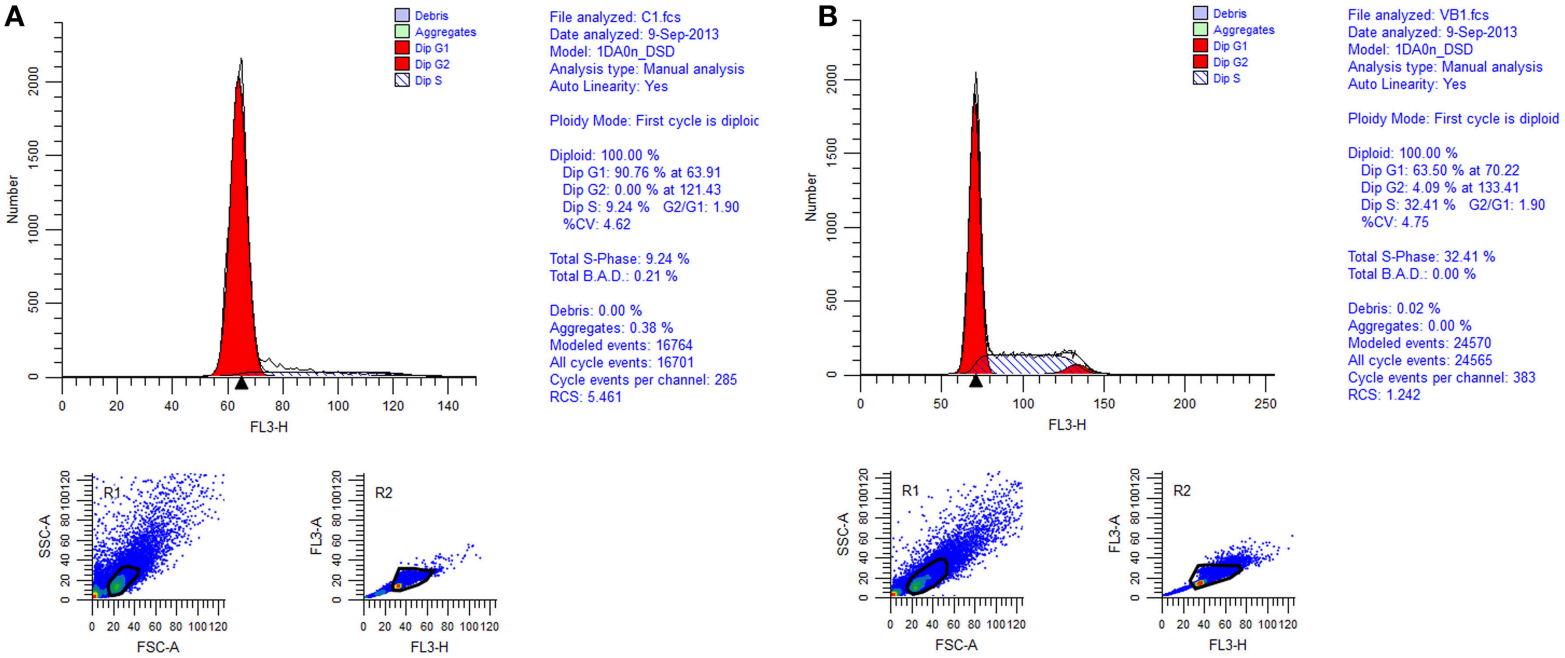
Cell cycle identification of cell by flow cytometry. **(A)** control cell; **(B)** REV infected cell.

**Figure 3:**
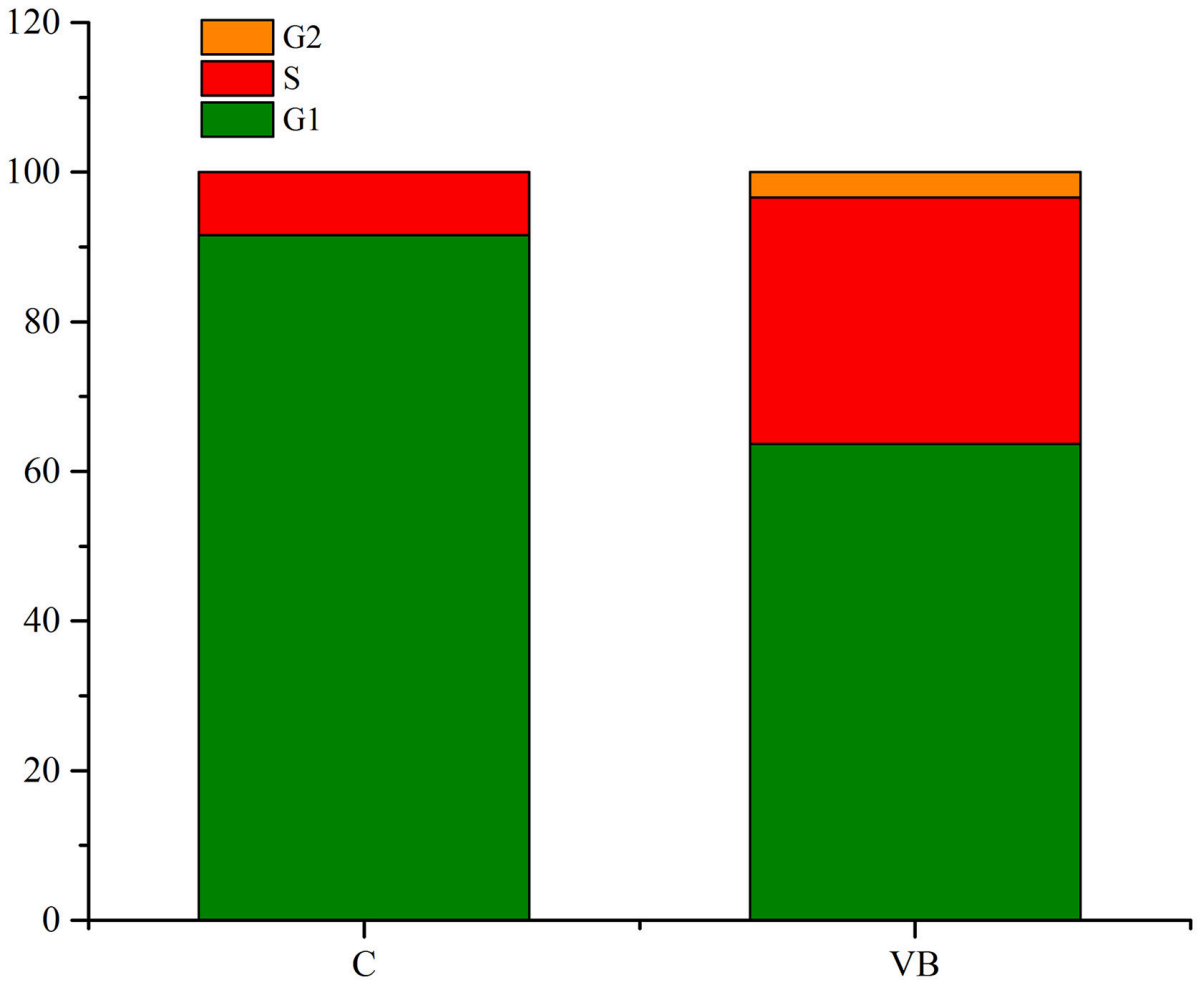
Value of G1/G2&S in control cell and REV infected cell. Y-axis represents the percent of total cells at cell phase.

Cell cycle analysis was conducted by flow cytometry and the results showed that 86.27% of the total control cells were in cell cycle of G_1_ phase, 13.71% of them were in S phase, and 0.02% of them were in G_2_ phase. While 54.76% of the total REV infected cells were in cell cycle of G_1_ phase, 39.69% of them were in S phase, and 5.55% of them were in G_2_ phase (Figures 2, 3). So, REV could promote the cell cycle of CEF cells.

### Illumina hiseq miRNA sequencing

**Figure 4:**
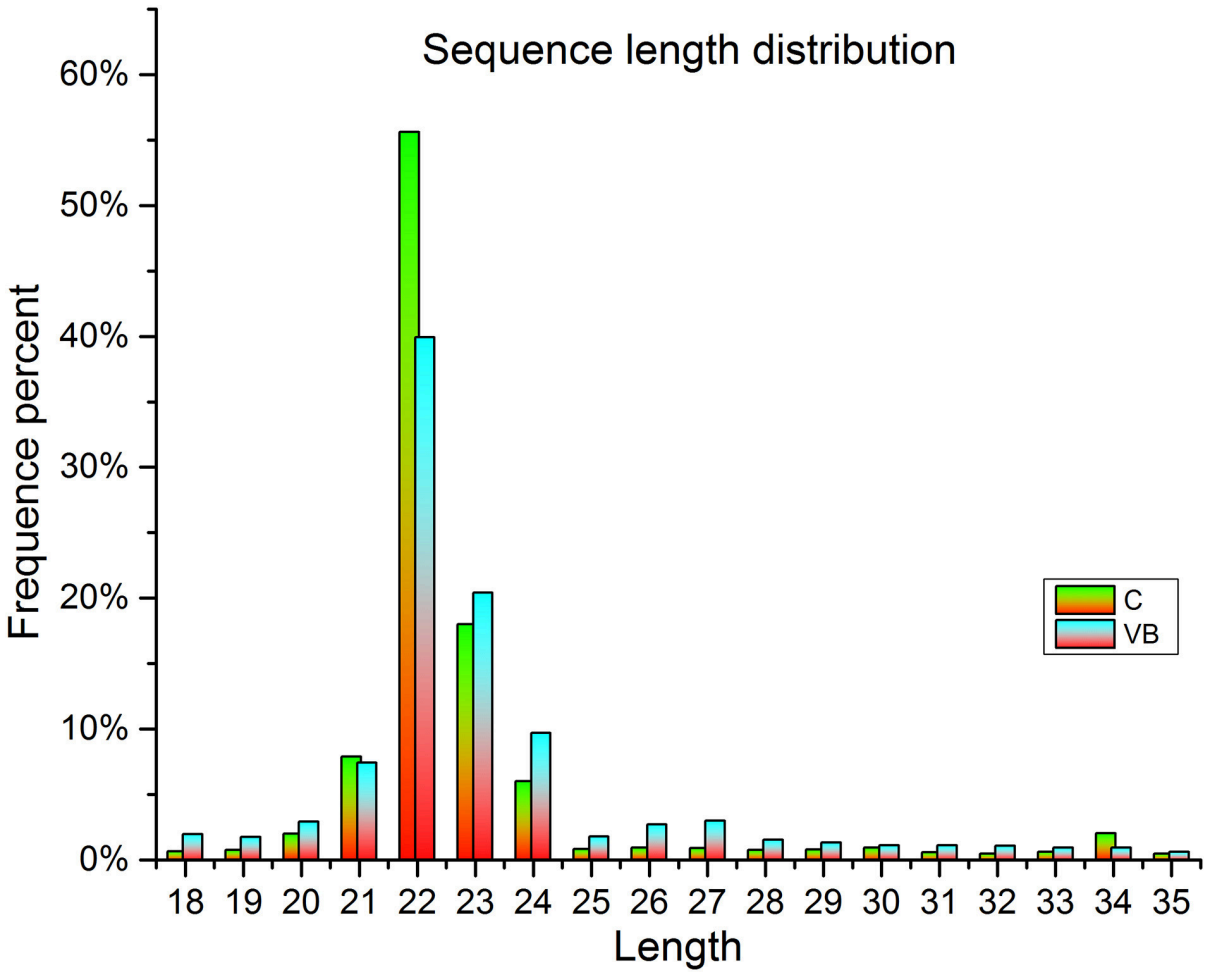
The length distribution of sequenced miRNAs in C (control) and VB.

In total, about 13.5 million raw reads were generated from the six libraries (Table 1), with about 13.1 million high-quality (*Q* > 20) short reads were selected for length filter (18–35 bp). After length filter, about 12.2 million reads remained for further study. 74.34–80.71% of the short clean reads were aligned against the “*Gallus gallus*” reference genome. Among which, 89.29–92.84% of filtered reads were mapped to gallus gallus reference genome which contains 63.23–50.8% known miRNA reads in control cells (280 reads) and VB cells (262 reads), respectively. Moreover, in total 431 known miRNAs were identified in the two samples including 370 reads in control cells, 393 reads in VB cells. Thirty novel miRNAs were predicted in the two samples including 24 in control cells, and 26 in VB cells (Table 2). The lengths of majority clean reads in both groups were 21–24 nt, while 22 nt RNAs were the most abundant which were consistent with the distribution pattern of miRNAs in previous studies [(Yang et al., 2014; Yu et al., 2017); Figure 4].

**Table 1:** Quality evaluation of raw reads.

**Sample**	**Reads**	**Bases**	**Error rate (%)**	**Q20 (%)**	**Q30 (%)**	**GC content (%)**
Control	6,170,894	0.309G	0.01	98.30	96.86	49.23
VB	7,326,817	0.366G	0.01	97.40	94.89	49.75

**Table 2:** Summary statistics of clean reads in CEF transcriptomes in different groups.

**Sample**	**Clean reads**	**Filtered reads**	**Mapped miRNA reads**	**Known miRNAs reads**	**Novel miRNA reads**	**Known miRNA number**	**Novel miRNA number**
Control	6,013,046	5,795,679	5,380,764 (92.84%)	3,402,429 (63.23%)	280	370	24
VB	7,148,295	6,380,115	5,696,644 (89.29%)	2,893,816 (50.8%)	262	393	26

**Figure 5:**
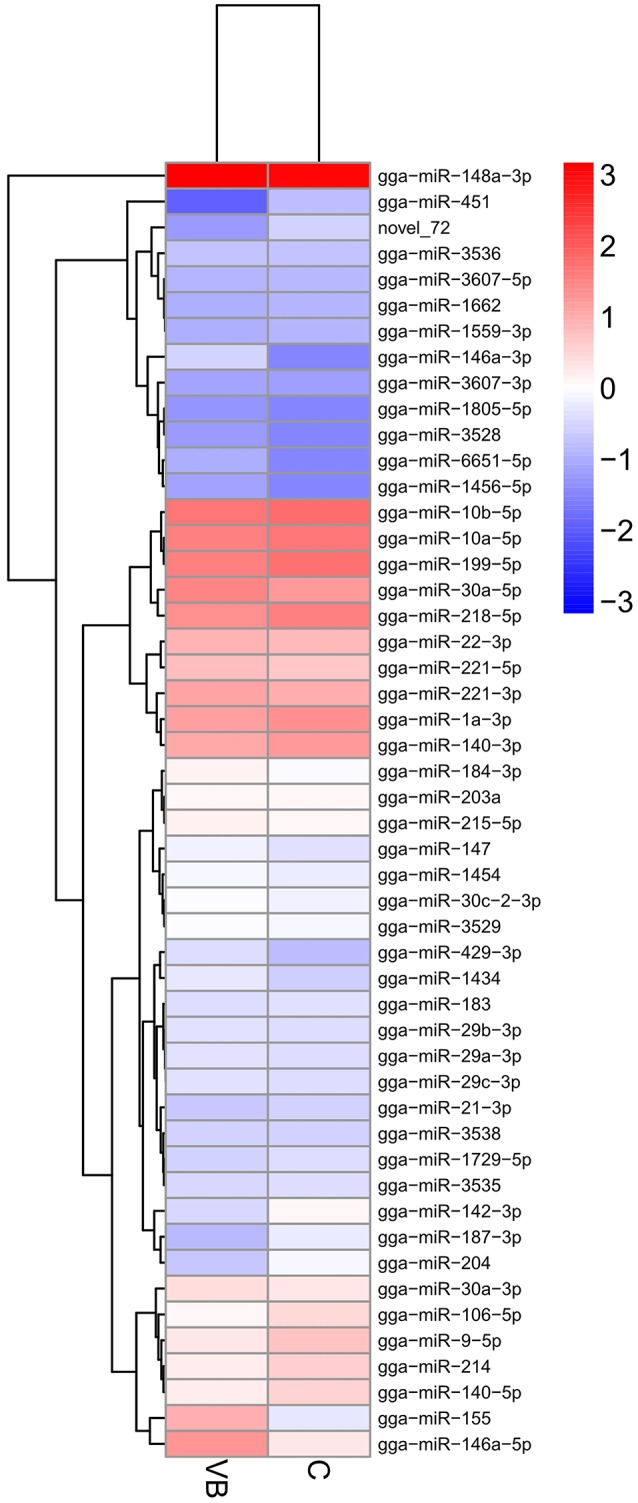
Heatmap of DEmiRNAs across two groups. Expression values of two libraries are presented as fpkm normalized log_10_ transformed counts.

Then DEmiRNAs of CEF cells of the two libraries were examined and were identified by setting *P* < 0.05 and fold change ≥1.5 as a default threshold. The result showed that there were 50 DEmiRNAs in VB group compared with control group including 34 significantly up-regulated and 16 significantly lower-expressed DEmiRNAs (Figure 5).

### Identification of miRNA target genes

**Figure 6:**
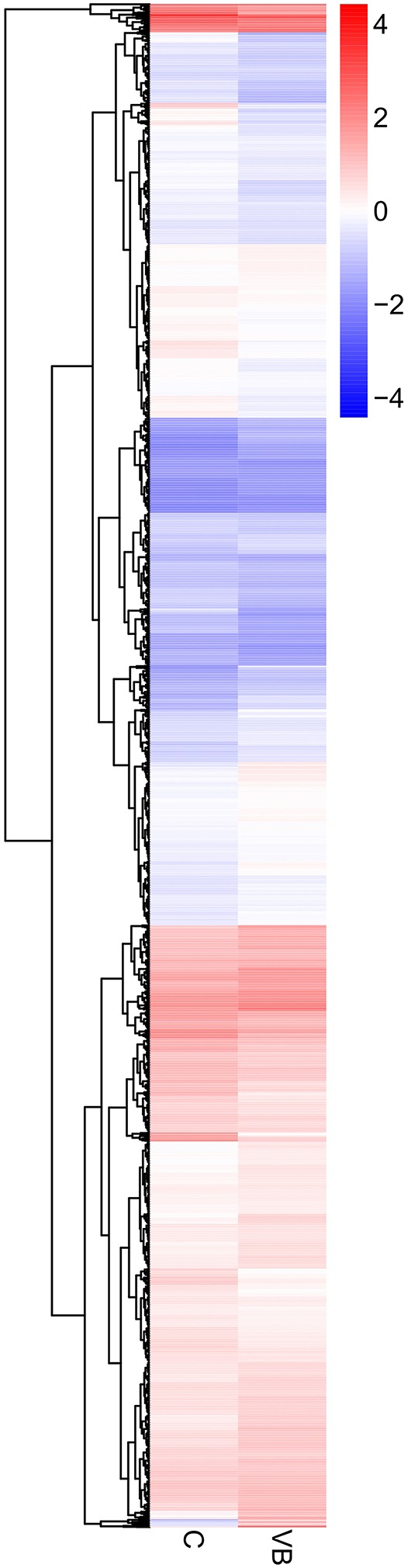
Heatmap and sub-clusters of DEGs across two groups Expression values of two libraries are presented as fpkm normalized log_10_ transformed counts.

Target genes of 50 DEmiRNAs were predicted and then identified by transcriptome results to exclude those predicted genes whose expression patterns are not in accordance with miRNA regulation. We tested that 3,203 differential expressed target genes (DEGs) were identified in VB group including 1,629 up-regulated DEGs were and 1,574 down-regulated DEGs (Figure 6).

**Figure 7:**
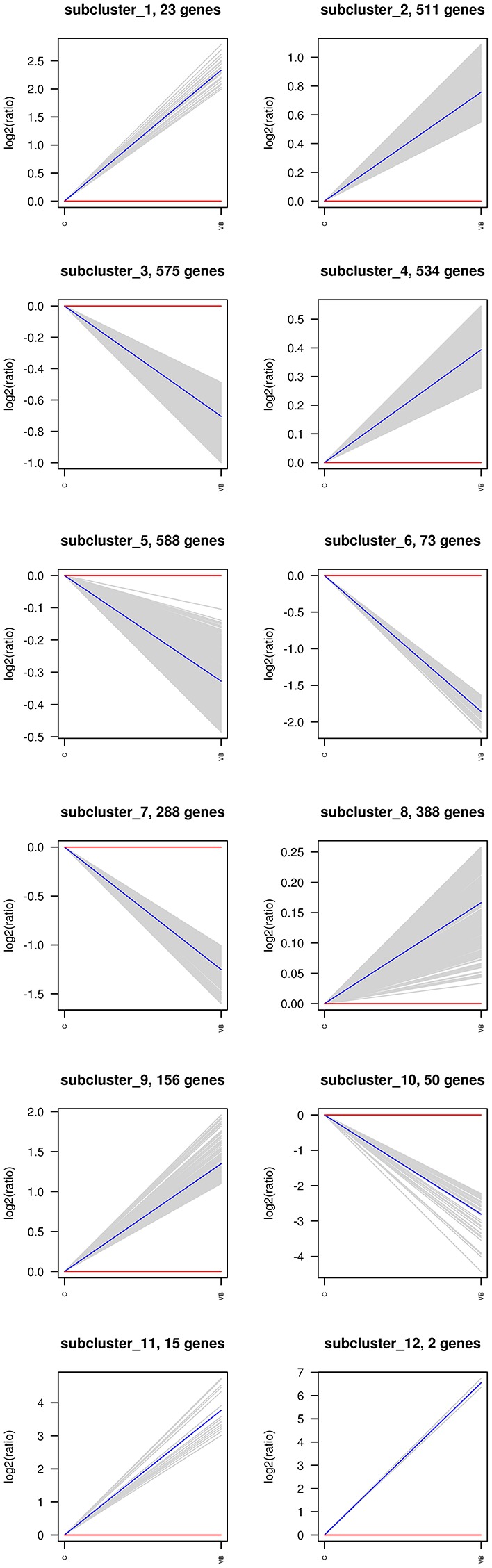
Same as Figure 6.

The hierarchical clustering of all the DEGs was performed using PHEATMAP SOFTWARE (*rkolde@gmail.com*). Twelve clusters were plotted with expression patterns. The sub1 and sub2 cluster include 23 and 511 genes which were up-regulated, whereas 575 genes in sub3 were down-expressed. Sub4, sub8, sub9, sub11 and sub12 contain 534, 388, 156, 15, and 2 over-expressed genes, respectively; while sub5, sub6, sub7, and sub10 contain 588, 73, 288, and 50 lower-expressed genes, respectively (Figure 7).

**Figure 8:**
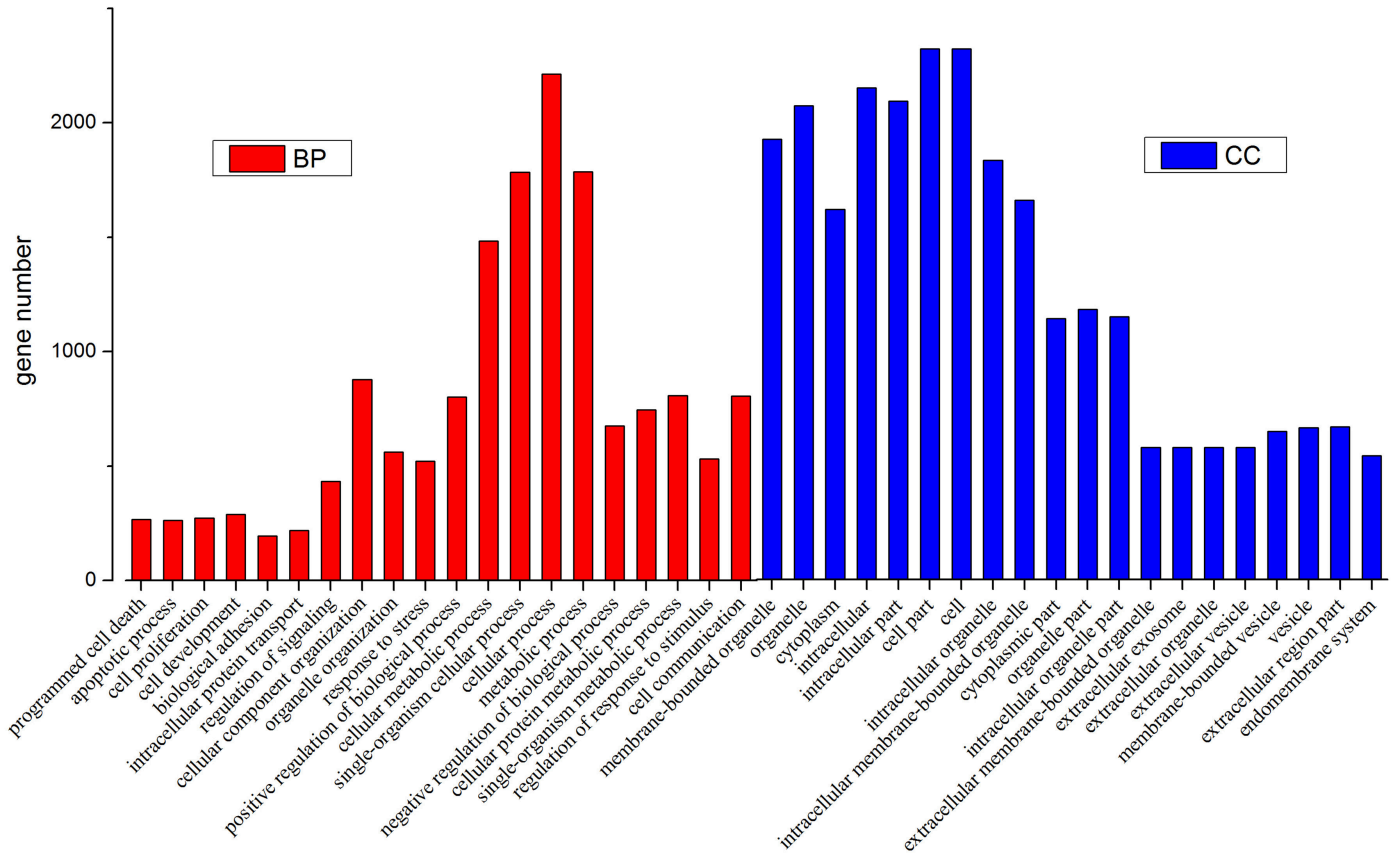
GO analysis of all the differentially expressed target genes of miRNAs.

**Figure 9:**
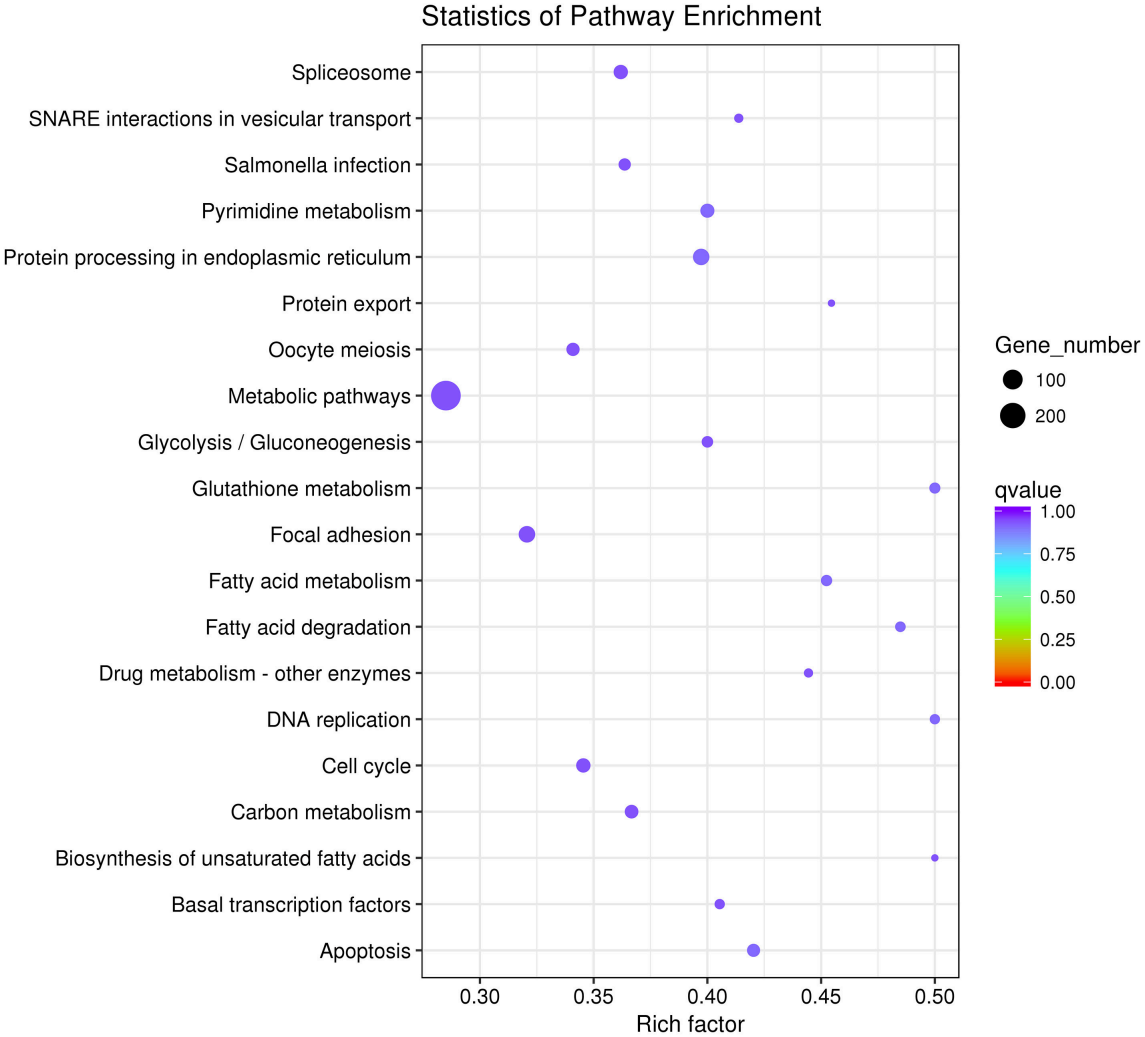
The top 20 enriched KEGG pathways of the differentially expressed target genes of miRNAs.

Then we used GO and KEGG analysis to predict the possible related biological processes and signaling pathways that play important roles in the infection of REV. GO analysis showed that DEGs of miRNAs function to regulate the biological process of programmed cell death, apoptotic process, cell proliferation, cell development, regulation of signaling, response to stress, metabolic process, cell communication, etc. (Figure 8). KEGG enrichment showed that cell cycle (gga04110) and apoptosis (gga04210) were the most enriched signaling pathways which were inconsistent with the former prediction (Yu et al., 2017) while most DEGs were enriched in metabolic pathway (Figure 9).

### Genes related to oncogenesis

**Figure 10:**
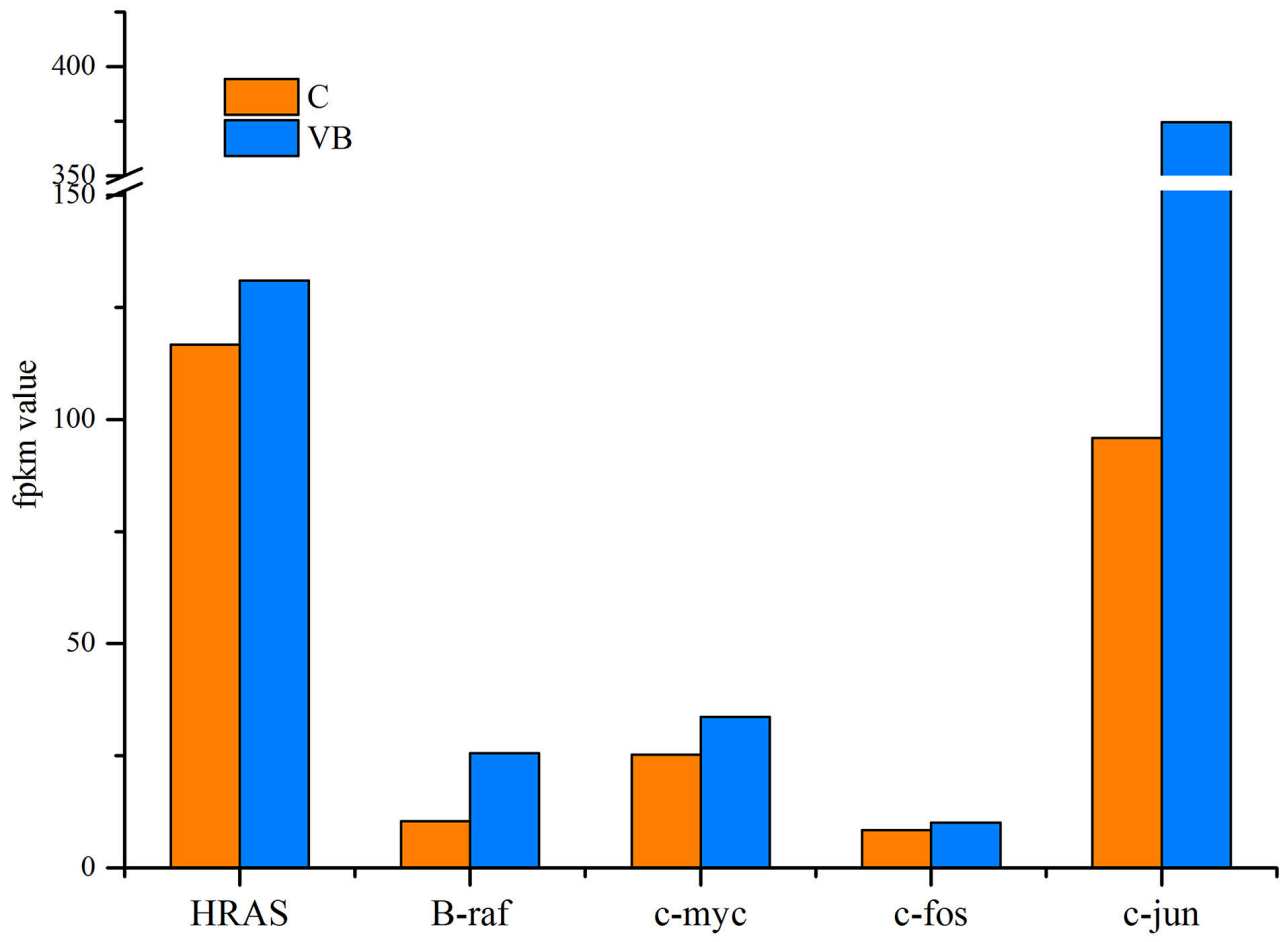
Expression level of oncogenes.

REV is a type of retrovirus that can cause lymphoma, so we identified genes related to cancer to figure out how REV can affect their expression. Our results showed that oncogenes including HRAS, B-Raf, c-myc, c-fos, and c-jun were all over expressed in REV infected cell. Since those genes over-expression or activation means the initiation of tumor formation, we expected that REV can activate the progress of oncogenesis of CEF cell (Figure 10).

### Regulation of cell cycle and apoptosis

**Figure 11:**
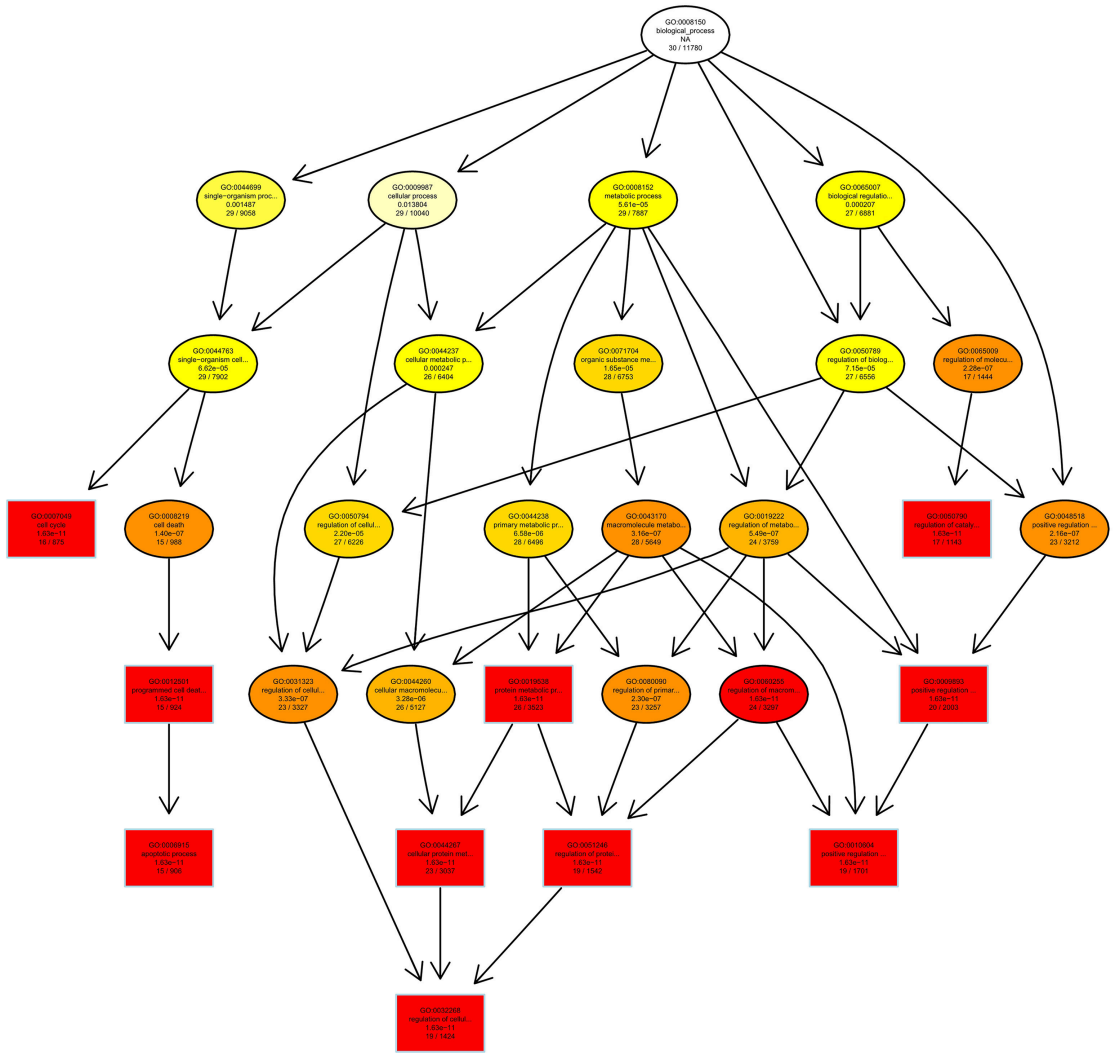
Directed Acyclic Graph of GO enrichment.

**Figure 12:**
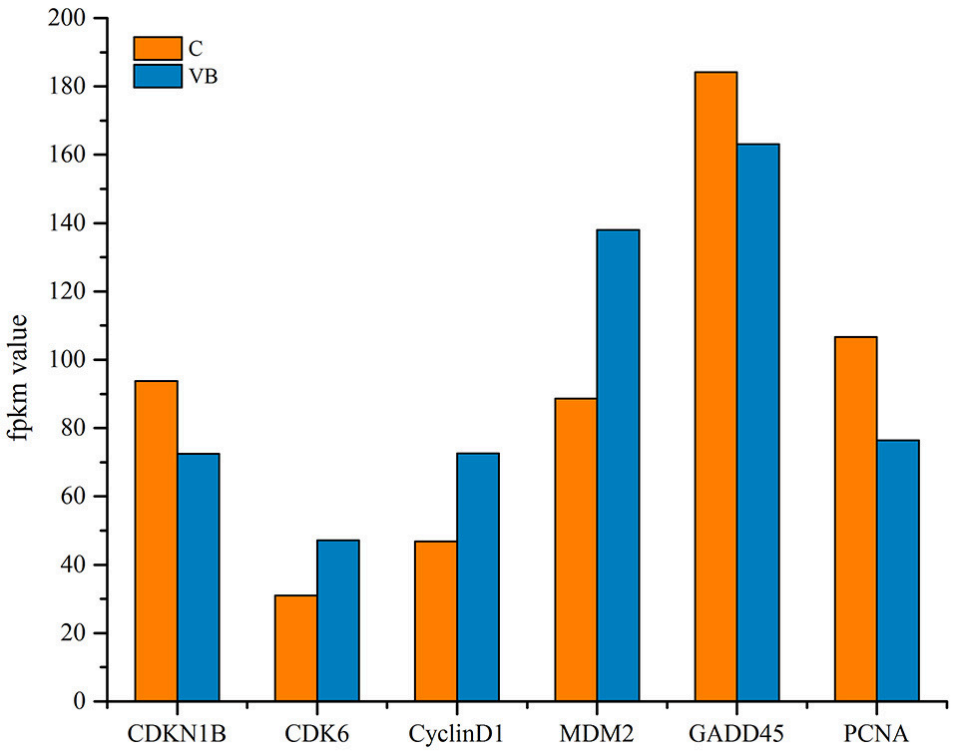
Expression level of genes related to regulation of cell cycle.

**Figure 13:**
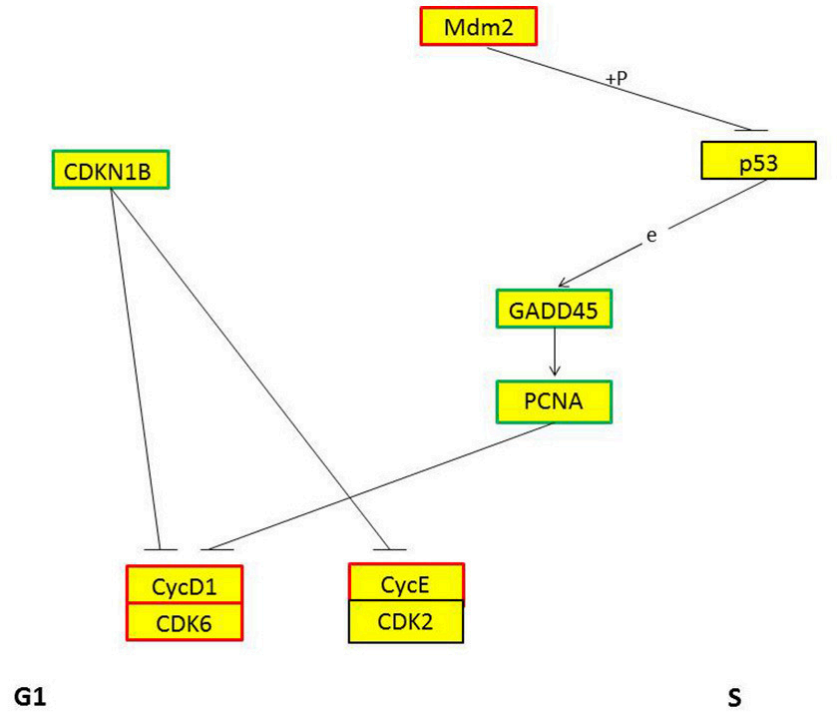
KEGG pathway of genes regulating cell period from G_1_ phase to S phase. Red frame means this gene is up-regulated, blue frame means this gene is down-regulated, + *p* means phosphorylation, e means expression.

GO and KEGG enrichment showed that cell cycle was one of the most enriched pathways in REV infected cell (Figures 8, 11). So, Genes, including CDKN1B, CDK6, CyclinD1, MDM2, GADD45, and PCNA were chosen to be analyzed. Among them, CDKN1B, GADD45, and PCNA were significantly down-regulated while CDK6, CyclinD1, and MDM2 were significantly over-expressed in REV infected cell than in control cell (Figure 12). KEGG enrichment showed that the up-regulation of MDM2 and the down-regulation of CDKN1B, GADD45, and PCNA promoted the over-expression of CyclinD1/CDK6 complex along the pathway (Figure 13). Since, CyclinD1/CDK6 complex plays a pivotal role in promoting the transition of the cell cycle from G1 phase to S phase, we concluded that REV control the cell cycle of CEF by pushing G1/S period which was in accordance with our cell cycle analysis.

**Figure 14:**
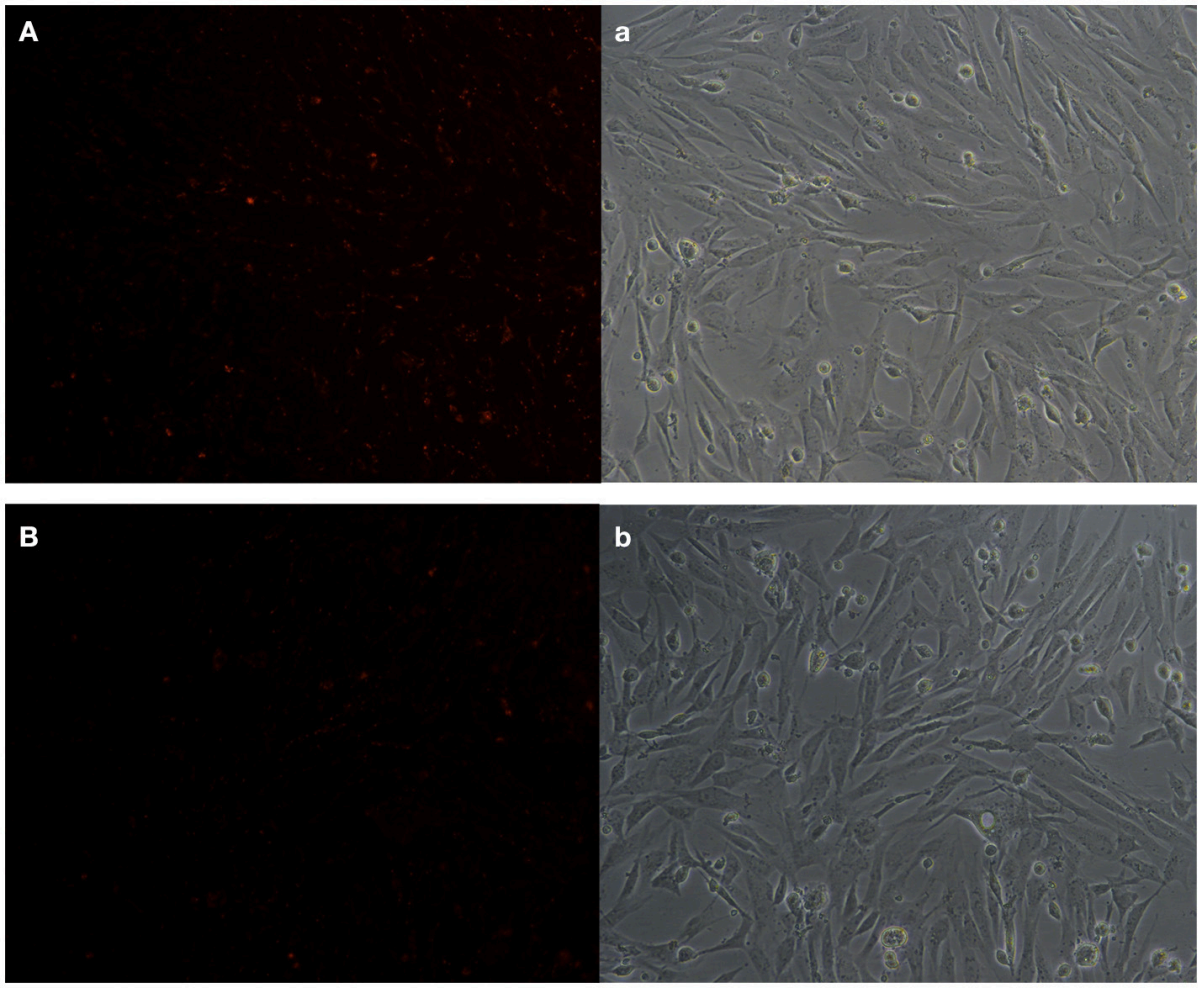
Apoptosis test result of CEF cells **(A)** control cell (**a**: bright field image of **A**), **(B)** REV infected cell (**b**: bright field image of **B**). We tested the mitochondrial membrane potential of two group cells; exposure time was set as 200 ms. The results showed that control cells were reacted with a higher fluorescent signal compared with REV infected cell, which meant REV can inhibit apoptosis of CEF.

**Figure 15:**
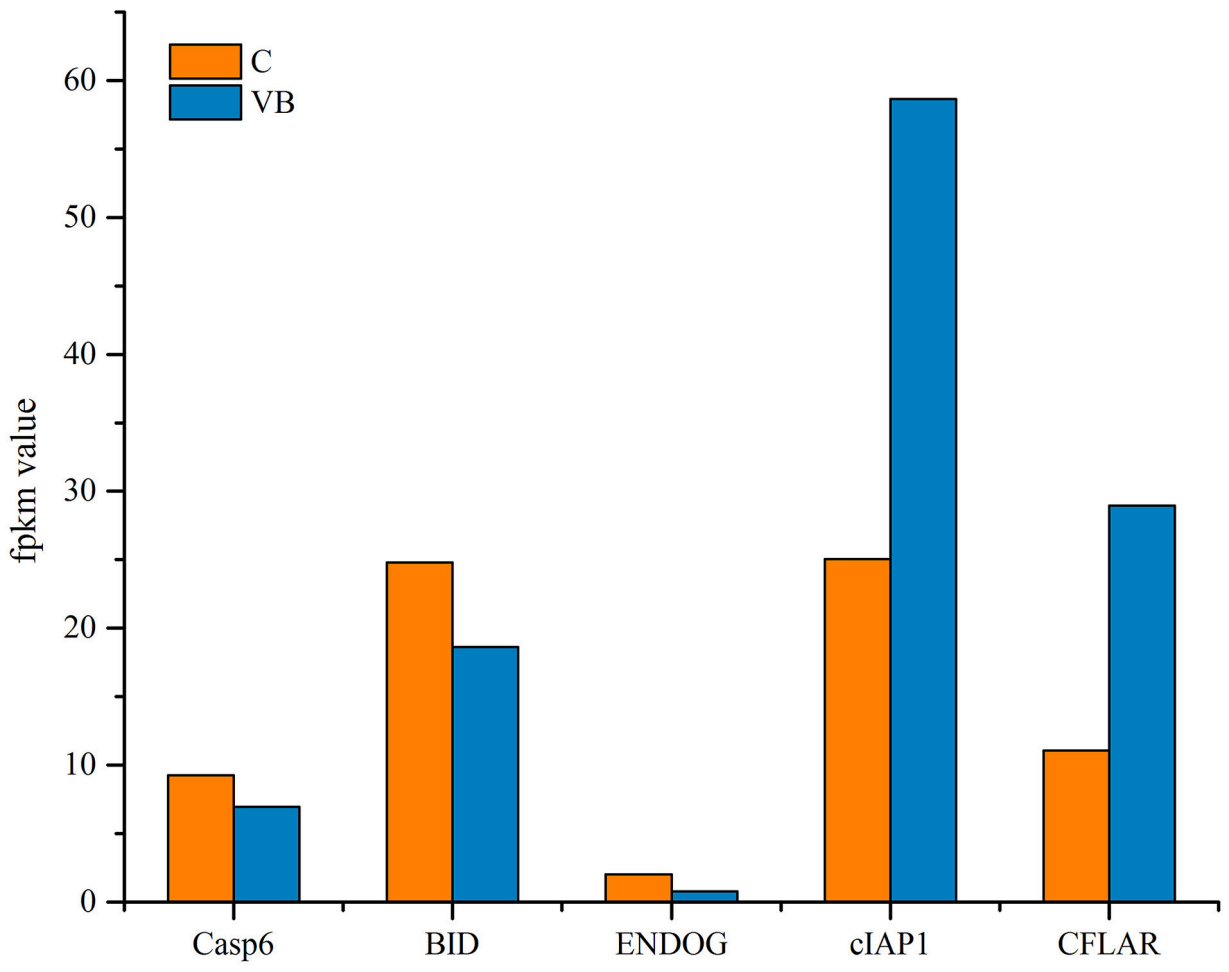
Target genes related to apoptosis.

KEGG analysis showed apoptosis is an enriched pathway. So, Fluorescence microscopy was applied to test the cell apoptosis. As shown in Figure 14, there were a stronger signal in control cells while a weaker signal in REV infected cells (Figure 14). This meant that REV inhibited the apoptosis of CEF cell. Then we analyzed the genes related to apoptosis to test whether those genes expression pattern is in accordance with the expected and this may help us to find new markers in the process of REV infecting its host. Five target genes including Casp6, BID, ENDOG, cIAP1, and CFLAR were analyzed. Among them, BID and ENDOG function to activate apoptosis while cIAP1 and CFLAR inhibit the activation of caspases to suppress apoptosis. In this study, cIAP1 and CFLAR showed an up-regulation in REV infected cell, while Casp6, BID, and ENDOG were down-regulated in REV infected cell which is in line with apoptosis analysis (Figure 15).

### Functional identification of novel-72

**Figure 16:**
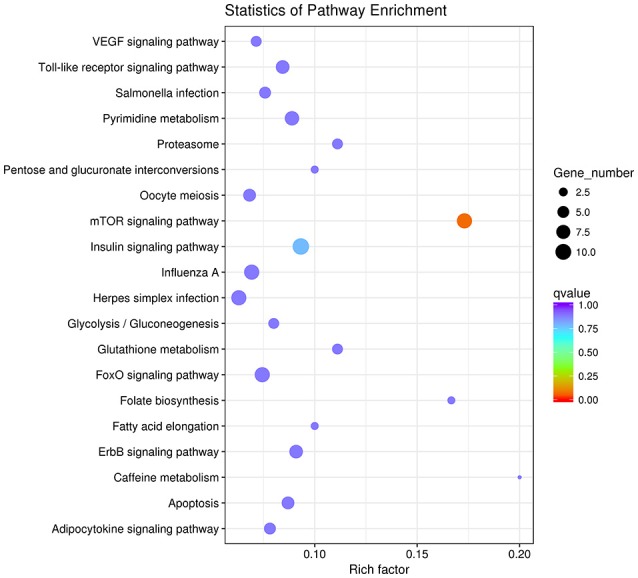
The top 20 enriched KEGG pathways of the differentially expressed target genes of novel-72.

**Figure 17:**
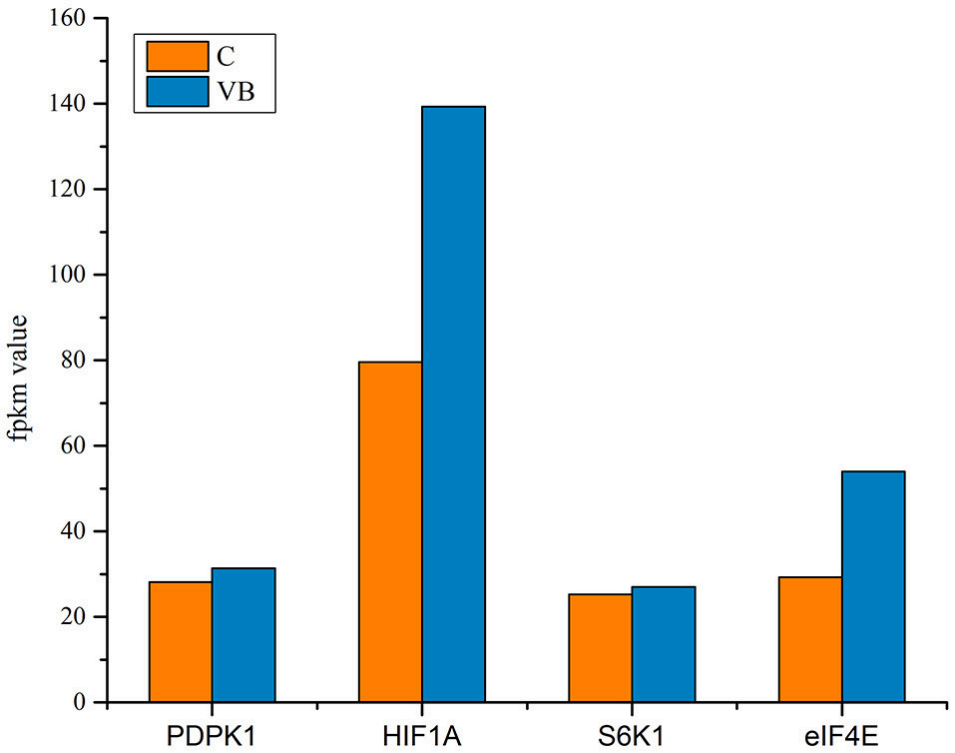
Genes related to mTOR signaling pathway.

We identified a new miRNA, novel-72 which showed a significant lower-expression in REV infected cell and KEGG enrichment analysis of its target genes showed that mTOR signaling pathway was most enriched (Figure 16). So, we identified the genes related to mTOR signaling pathway to analyze the possible functions of novel-72 and the role of mTOR pathway in REV infection. Four genes which were previously reported to be involved in cell proliferation and cell survival including PDPK1, mTOR, S6K1, and eIF4E were analyzed. Transcriptome sequencing analysis showed that all of the four genes were significantly elevated in REV infected cell (Figure 17), so novle-72 control the cell fate mediated by mTOR signaling pathway in the pathogenesis of REV infection.

### Verification of miRNAs and the target genes expression using RT-qPCR

**Figure 18:**
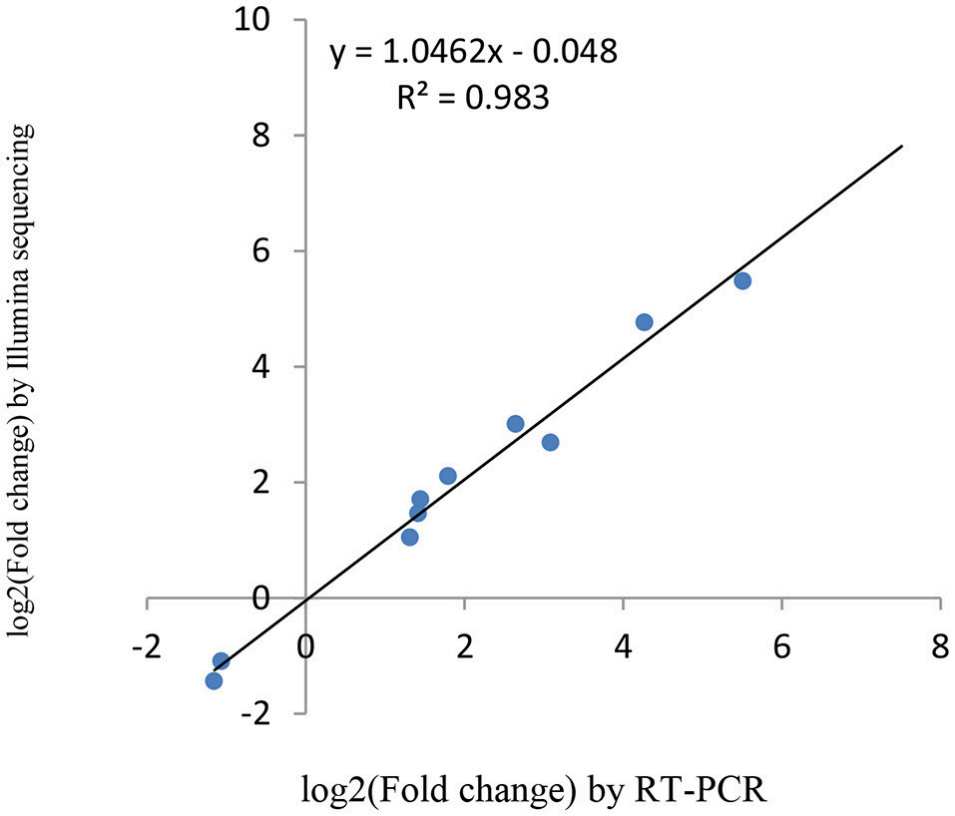
qRT-PCR validation of differentially expressed miRNAs.

**Figure 19:**
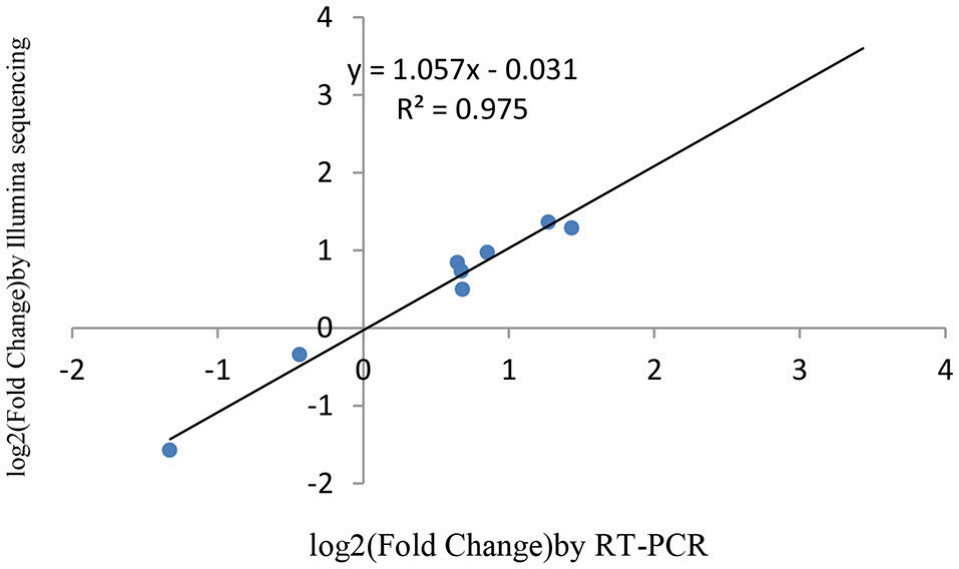
qRT-PCR validation of differentially expressed mRNA targets.

**Figure 20:**
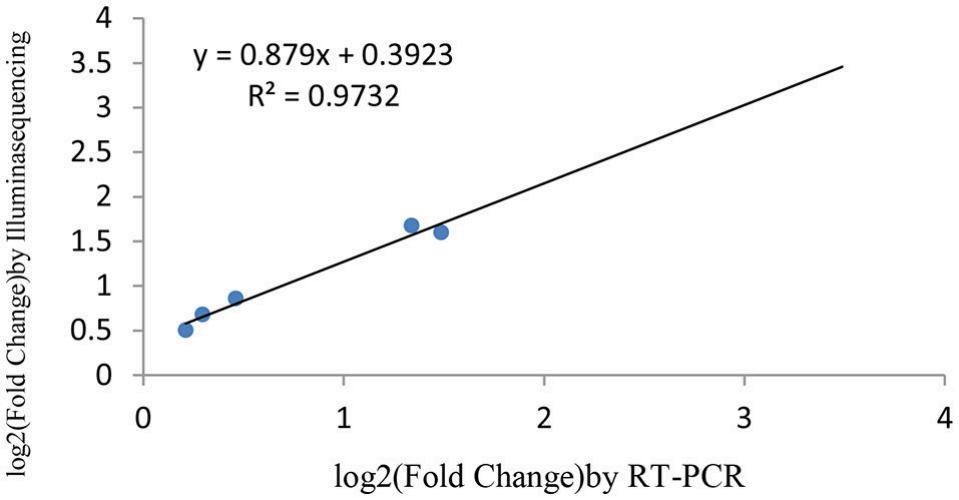
qRT-PCR validation of the five oncogenes.

miRNAs and its transcriptional regulation revealed by RNA-seq were confirmed in a biologically independent experiment using RT-qPCR. A total of 21 miRNAs and 19 target genes including 6 genes involved in cell cycle, 5 genes related to apoptosis, and 8 genes involved in mTOR signaling pathway were chosen to design gene/miRNA specific primers (Tables S1, S2). The fold changes in expression of 10 differentially expressed miRNAs, eight differentially expressed target genes and five oncogenes were comparable between the two datasets, with correlation coefficients of *R*^2^ = 0.983, 0.975 and 0.9732 (*P* < 0.001), respectively. Taken together, these results suggest that the high-throughput sequencing results in this study were reproducible (Figures 18–20).

## Discussion

Previously, we have identified miRNAs expression profile of bursa of *Fabricius* in SPF chickens in response to REV infections and discovered that miRNAs not only involved in the up-regulation of genes related to proapoptotic, proto-oncogene, and carcinogenic cytokines, but also involved in down-regulation of genes related to anti-apoptotic cytokines (Yu et al., 2017). By using microarray analysis, Ji Miao analyzed the transcripotme of CEF and found that genes involved in immune response played important roles in the pathogenicity of REV infection (Ji et al., 2015). Here we identified five significantly over-expressed oncogenes including HRAS, B-Raf, c-myc, c-fos, and c-jun. The abnormal expression of those genes strongly implicated in cancer (Vogt, 2002; Milde-Langosch, 2005; Kessler et al., 2012). Even though REV could not induce cancer in CEF, this virus may activate the identical mechanism in CEF that cause lymphoma in its host. Since we tested a promotion of cell cycle and an inhibition of apoptosis in REV infected CEF, we believe REV can activate the mechanisms of tumorigenesis of CEF. So, identifying the exact target genes of miRNAs in CEF and figure out the functional miRNAs are meaningful to help us figure out how REV interacts with its host.

As a retrovirus, REV could integrate its genes into the cell genome to affect its host. miRNAs may be as a regulator in pathogenesis of REV infection. KEGG enrichment showed that target genes of some miRNAs were enriched in the cell cycle. In accordance with KEGG enrichment analysis, we found a higher ratio of S phase cells while a lower ratio of G_1_ phase cells in REV infected cell than in control cell indicating that REV could promote the progression of CEF cell cycle. Then target genes related to the transition from G_1_ phase to S phase were tested. CDKN1B gene targeted by miRNAs prevents the activation of cyclinD-CDK complexes that controls the cell cycle progression at G1, which showed a lower expression thus may lead the up-regulation of CDK6 and CyclinD1. CDK6 and CyclinD1were also targeted by miRNAs which may directly lead to the up-regulation of these two genes to control the cell cycle. Mdm2 is a target gene of miRNAs, and KEGG analysis showed that it can up-regulate CyclinD1-CDK6 by inhibiting GADD45 and PCNA through p53 signaling pathway (Figure 13). Therefore the CyclinD1-CDK6 complex is important in the transition of the cell cycle from G1 phase to S phase during REV-CEF interaction.

We investigated the inhibition of apoptosis in REV infected cells. Target genes related to apoptosis were analyzed to identify the activity of the functional genes and miRNAs during the progress of REV infections. Both CFLAR and cIAP1 expression are up-regulated. These two genes are members of the inhibitor of apoptosis family that inhibit apoptosis by interfering with the activation of caspases (Irmler et al., 1997; Samuel et al., 2006). The up-regulation of them likely suppressed the expression and activation of other caspases and led to the down-regulation of caspase6 which acted as an executor of cell death. Caspase6 is also the target genes of miRNAs which suppressed the expression of Caspase6 to restrain the progression of apoptosis. Therefore, miRNAs may control Caspase6 directly or by regulating CFLAR and cIAP1 to affect apoptosis. The BID gene is a pro-apoptotic member of a Bcl-2 protein family, its down-regulation prevented the release of pro-apoptotic factors like ENDOG that function to promote apoptosis. By regulating BID expression, the ENDOG could be controlled to prevent its degradation of DNA to prevent apoptosis. Thus, REV can inhibit apoptosis to contribute to the promotion of CEF cell cycle and this may be the potential mechanism that REV cause tumorigenesis.

We identified a new miRNA, novel-72 and its target genes were most enriched in mTOR signaling pathway. In this study, the targeted S6K1 and eIF4E were significantly up-regulated in REV infected cell. Fingar et al. (2004) have found that the S6K1 and eIF4E pathways positively regulated G1 phase progression which is in accordance with our results of cell cycle test. So, Novel-72 may regulate the cell transition from G1 phase to S phase by targeting those two genes. HIF1A functions to regulate cellular metabolism to overcome hypoxia, while hypoxia promotes apoptosis in both normal and tumor cells. So, in this study HIF1A played a role in inhibiting cell apoptosis. Also, HIF1A can activate VEGF signaling pathway to target genes like Ras and Raf to regulate cell proliferation. Reports showed that the PDPK1/AKT/mTOR pathway is an intracellular signaling pathway important in regulating the cell cycle (Rafalski and Brunet, 2011; King et al., 2015). Then another possibility is that novel-72 targeted PDPK1 which then activated mTOR signaling pathway to regulate the expression of S6K1, eIF4E, and HIF1A that controlled the cell fate. Here what we can confirm is that novel-72 is important in cell cycle and cell proliferation in the pathogenesis of REV infection.

## Author contributions

JZ as the first author, designed and conducted the main experiments, and accomplished the writing of this article. CG and LF helped analyzing the data. LJ and SD helped do the part of RT-PCR experiment. SZ supervised the whole program of the experiments.

### Conflict of interest statement

LJ was employed by company CTI Biotechnology (Suzhou) Co., Ltd. The remaining authors declare that the research was conducted in the absence of any commercial or financial relationships that could be construed as a potential conflict of interest.
