# Supplementary material for: Integrative Analyses of Transcriptome Sequencing Identify Functional miRNAs in the Chicken Embryo Fibroblasts Cells Infected With Reticuloendotheliosis Virus
Source: Front Genet. 2018 Aug 29;9:340. doi: 10.3389/fgene.2018.00340 (PMC6128223; doi:10.3389/fgene.2018.00340)
Supplement: Supplementary file 1 [file Table_1.docx]

Appendixes

TableS1 Primers used to assay the target genes expression by RT-qPCR

| Gene name | Forward primer (5'-3') | Reverse primer (5'-3') | Length |
| --- | --- | --- | --- |
| CDKN1B | GCTTCCCGACTTCTACTTC | TGTCCTCTGAGATTCCCTG | 135 |
| CyclinD1 | TTCCACTAACACTTTCCTCTC | GGTCTGCTTCGTCCTCTAC | 184 |
| CDK6 | GATTGACTCCCAGAAGAAGA | GTGAGACAGGGCAGCATAG | 188 |
| MDM2 | GAGTCCAGTCTGCCTGTTAG | GTCCTCTTCTTCAGTTTTCTT | 137 |
| GADD45 | TTAGCTCAGCGTCGGG | TTGCACGATGTGGATGTC | 175 |
| PCNA | TAAGCAAATCAGGAAAAGGT | CACAGGAGATGACAACAGC | 175 |
| CASP6 | GTTTGTTTGTGTGTTCTTGAGT | GGTTATCTTTGGCTTTCCTA | 134 |
| BID | TCACAGGCAGTGGAAGG | TTGTGTTGGCTGATGTAGTT | 166 |
| ENDOG | GTCATCGAGCAGCTCAACC | ATGCTTCTGCTGTATTTCTC | 257 |
| cIAP1 | AGCATCGGAGGCACTTTC | GCTGAACTGGAATCCGAGT | 146 |
| CFLAR | ACGCCCTTGAAAGACACT | ACCAAACAACGCAGATAATA | 120 |
| PDPK1 | GTGTGGTGTTGTGTTCCTG | TATCCCTGTCTGCTGCC | 100 |
| HIF1A | TTGTTCCATCATCTCCTGTC | CTGTTCCAATGTTCCTTTTC | 122 |
| S6K1 | AGGAGTGGGCATAATCGT | ATGGCTTCTTGTGTGAGGT | 153 |
| eIF4E | TCACTAACCAAACAGCAGAG | AACAACAGCACCACATACAT | 117 |
| AKT1 | TTGAGTTGTTTTTCCATCTGT | TTATGTGCCCGTCTTTATC | 167 |
| TSC1 | TCTCTCTTTCATCGGCTTT | ATTGGCTTGACTACCTCTTC | 113 |
| TSC2 | TGTGGGTGAGTTTCTGTTG | GTCTGAGCCTGATTCTGTG | 155 |
| mTOR | TCCCTTATCCTCACCACTC | TCACGGTTCATTCCTTTCT | 128 |

Table.S2 Primers used for RT-qPCR identification of miRNAs

| miRNA ID | Primers(5'-3') | Length |
| --- | --- | --- |
| gga-miR-21-3p  gga-miR-22-3p  gga-miR-29a-3p  gga-miR-29c-3p  gga-miR-30a-3p  gga-miR-30a-5p  gga-miR-30c-2-3p  gga-miR-106-5p  gga-miR-140-3p  gga-miR-140-5p  gga-miR-142-3p  gga-miR-146a-5p  gga-miR-148a-3p  gga-miR-155  gga-miR-183  gga-miR-203a  gga-miR-204  gga-miR-214  gga-miR-218-5p  gga-miR-221-3p  gga-miR-221-5p  novel-72  U6 | RT: GTCGTATCCAGTGCAGGGTCCGAGGTATTCGCACTGGATACGACGACAGC  F: GGCAACAACAGTCGGTAG  RT: GTCGTATCCAGTGCAGGGTCCGAGGTATTCGCACTGGATACGACACAGTT  F: GCTTCGACGGTCAACTTC  RT: GTCGTATCCAGTGCAGGGTCCGAGGTATTCGCACTGGATACGACAACCGA  F: CTCGTAGCACCATTTGAAA  RT: GTCGTATCCAGTGCAGGGTCCGAGGTATTCGCACTGGATACGACACCGAT  F: CTCGATAGCACCATTTGAAA  RT: GTCGTATCCAGTGCAGGGTCCGAGGTATTCGCACTGGATACGACGCTGCA  F: CGAAAGTCAGCCTACAAAC  RT: GTCGTATCCAGTGCAGGGTCCGAGGTATTCGCACTGGATACGACCTTCCA  F: CGGACATTTGTAGGAGCT  RT: GTCGTATCCAGTGCAGGGTCCGAGGTATTCGCACTGGATACGACAGAGTA  F: TGTGGGAGAAGGCTGTTT  RT: GTCGTATCCAGTGCAGGGTCCGAGGTATTCGCACTGGATACGACTACCTG  F: GCCTTTTCACGAATGTCAC  RT: GTCGTATCCAGTGCAGGGTCCGAGGTATTCGCACTGGATACGACGTCCGT  F: GCGGTGTCCCATCTTG  RT: GTCGTATCCAGTGCAGGGTCCGAGGTATTCGCACTGGATACGACCTACCA  F: GCCTCACCAAAATGGG  RT: GTCGTATCCAGTGCAGGGTCCGAGGTATTCGCACTGGATACGACCCATAA  F: AGGGCTGTAGTGTTTCCTACT  RT: GTCGTATCCAGTGCAGGGTCCGAGGTATTCGCACTGGATACGACAACCCA  F: CTGCAGAGAACTGAATTCCA  RT: GTCGTATCCAGTGCAGGGTCCGAGGTATTCGCACTGGATACGACACAAAG  F: CCGTCAGTGCACTACAGA  RT: GTCGTATCCAGTGCAGGGTCCGAGGTATTCGCACTGGATACGACCCCCTA  F: ACGGTTAATGCTAATCGTGA  RT: GTCGTATCCAGTGCAGGGTCCGAGGTATTCGCACTGGATACGACCAGTGA  F: GCCATACCGTGACCATCT  RT: GTCGTATCCAGTGCAGGGTCCGAGGTATTCGCACTGGATACGACCAAGTG  F: GCCCACTTTACAAATCCT  RT: GTCGTATCCAGTGCAGGGTCCGAGGTATTCGCACTGGATACGACAGGCAT  F: CGAAGGGAAACAGTAGGAT  RT: GTCGTATCCAGTGCAGGGTCCGAGGTATTCGCACTGGATACGACCTGCCT  F: TGACAGCAGGCACAGACA  RT: GTCGTATCCAGTGCAGGGTCCGAGGTATTCGCACTGGATACGACACATGG  F: GGCCAACACGAACTAGAT  RT: GTCGTATCCAGTGCAGGGTCCGAGGTATTCGCACTGGATACGACGAAACC  F: GCCTCGATGTAACAGACG  RT: GTCGTATCCAGTGCAGGGTCCGAGGTATTCGCACTGGATACGACACAGAA  F: GCCTTGGACCGTATGTT  RT: GTCGTATCCAGTGCAGGGTCCGAGGTATTCGCACTGGATACGACAATTCA  F: GGCGTCCTTGTTGACTTC  R: GTGCAGGGTCCGAGGT  F: GGAACGATACAGAGAAGATTAGC  R: TGGAACGCTTCACGAATTTGCG | 50  18  50  18  50  19  50  20  50  19  50  18  50  18  50  19  50  16  50  16  50  21  50  20  50  18  50  20  50  18  50  18  50  19  50  18  50  18  50  18  50  17  50  18  16  23  22 |
